# Exhaled Biomarkers in Idiopathic Pulmonary Fibrosis—A Six-Month Follow-up Study in Patients Treated with Pirfenidone

**DOI:** 10.3390/jcm9082523

**Published:** 2020-08-05

**Authors:** Kaja Jaskiewicz, Katarzyna Mycroft, Marta Maskey-Warzechowska, Karolina Paralusz, Natalia Siemiez, Patrycja Nejman-Gryz, Malgorzata Barnas, Rafal Krenke, Katarzyna Gorska

**Affiliations:** 1 Department of Internal Medicine, Pulmonary Diseases and Allergy, Medical University of Warsaw, 02-091 Warszawa, Poland; kaja.jaskiewicz@uckwum.pl (K.J.); katarzyna.mycroft@wum.edu.pl (K.M.); marta.maskey@wum.edu.pl (M.M.-W.); patrycja.nejman-gryz@wum.edu.pl (P.N.-G.); mbarnas@wum.edu.pl (M.B.); rafal.krenke@wum.edu.pl (R.K.); 2 Students’ Research Group “Alveolus”, Medical University of Warsaw, 02-091 Warsaw, Poland; karolina.paralusz@onet.pl (K.P.); nataliasiemiez@gmail.com (N.S.)

**Keywords:** antifibrotic treatment, exhaled breath condensate, pulmonary fibrosis, interleukin, vascular endothelial growth factor

## Abstract

The mechanism of action of pirfenidone in idiopathic pulmonary fibrosis (IPF) has not been fully elucidated. To offer additional insight, we evaluated the change in the cytokine profile in exhaled breath condensate (EBC) following a six-month treatment with pirfenidone in patients with IPF. EBC concentrations of interleukin (IL)-6, IL-8, IL-15, TNF-α and VEGF-A were assessed with ELISA and compared at baseline and after six months of pirfenidone treatment. Twenty-nine patients with IPF and 13 controls were evaluated at baseline. With the exception of IL-8 concentration, which was lower in patients with IPF when compared to controls (*p* = 0.005), the cytokine levels did not differ between the groups. Despite the use of a high sensitivity assay, IL-8 reached detectable values only in 24% of IPF patients. EBC analysis after six months of treatment with pirfenidone did not reveal any differences in the cytokine levels. The change in EBC vascular endothelial growth factor A (VEGF-A) correlated with the change in the 6 min walk distance (r = 0.54, *p* = 0.045). We conclude that a six-month treatment with pirfenidone did not significantly change the EBC cytokine profile. Our findings support the potential usefulness of VEGF-A as a marker in IPF. The low EBC IL-8 level in patients with IPF is a novel finding which needs confirmation in larger studies.

## 1. Introduction

Idiopathic pulmonary fibrosis (IPF) is a chronic progressive fibrosing interstitial lung disease of unknown etiology, which occurs primarily in older adults. Although the trigger for IPF is unknown, lung fibrosis in IPF seems to be regulated by a complex network of mediators [1,2]. It is currently hypothesized that IPF is a consequence of repeated microinjuries to the alveolar epithelium, which lead to dysregulation of wound healing and causes fibrosis by activating myofibroblasts [3]. Both the innate and adaptive systems appear to be involved in IPF. Macrophages are one of the most studied innate immune cells in IPF pathogenesis. They can contribute to fibrosis by secreting immune molecules, such as profibrotic tumor necrosis factor-alpha (TNFα), interleukin (IL)-1, IL-6, IL-8 and vascular endothelial growth factor (VEGF), which can both promote or inhibit the cascade of fibrosis [2]. IL-6 can stimulate fibrogenesis either alone or with TNFα, and levels of IL-6 were significantly higher in bronchoalveolar lavage fluid (BALF) from patients with IPF in comparison to control subjects [1]. Some studies have indicated an important role of IL-8. Elevated levels of IL-8 were found in serum, sputum and BALF [4,5,6]. Moreover, high blood IL-8 concentrations were shown to be associated with disease activity [7] and increased mortality in IPF [8]. 

There are limited treatment options in IPF to date. Systemic steroids and immunosuppressive drugs had very modest efficacy, did not improve outcomes and increased the risk of death [9]. In recent years, new medications, i.e., pirfenidone and nintedanib, were introduced and were shown to decelerate the decline in forced vital capacity [10], slow the progression of dyspnea [11] and to improve survival [12]. Pirfenidone is a simple compound of a ring-like structure, a pyridine derivative (5-methyl-phenyl-2-(1H)-pyridone) with antifibrogenic, anti-inflammatory and antioxidant effects [13]. This effect has been demonstrated in both in vitro and in vivo studies. It has been proven that pirfenidone reduces collagen synthesis, blocks the proliferative activity of platelet-derived growth factor (PDGF) in hepatocytes, reduces proliferation of fibroblasts and reduces pro-collagen concentration [13,14]. In in vivo models, pirfenidone reduces the synthesis of fibrogenic cytokines such as TNFα, IFNγ and IL-6 [15]. Animal models of lung fibrosis (bleomycin-induced fibrosis) have shown the efficacy of pirfenidone in inhibiting the development of fibrosis, reducing oxidative stress indicators and reducing neutrophilic inflammation [16,17]. There is a limited number of such studies in humans to date. Ronan et al. observed an increase in angiogenesis cytokine levels and a decrease in proinflammatory cytokines in bronchoalveolar lavage fluid after six months of pirfenidone treatment [18]. However, bronchoalveolar lavage can be obtained only via bronchoscopy, which carries a significant risk in patients with severe disease or multimorbidity and may trigger IPF exacerbation [19]. 

Exhaled breath condensate (EBC) is a noninvasively collected matrix from the respiratory tract, which contains various inflammatory mediators. Majewski et al. demonstrated that interleukin 25 and 33 are detectable in EBC from patients with IPF and that their concentrations show some significant correlations with lung function in these patients [20]. We hypothesized that EBC might be a promising respiratory sample to provide additional insight into the mechanisms involved in the pathogenesis of IPF and to monitor antifibrotic treatment. Thus, we undertook an exploratory study aimed to assess the usefulness of EBC in the evaluation of treatment response in patients receiving antifibrotic therapy. The specific aims of the study were: 1/ to assess the feasibility of selected cytokine concentration measurements in EBC from patients with IPF, 2/ to compare the EBC cytokine profile in patients with IPF and controls, and, 3/ to compare EBC cytokine concentrations in patients with IPF at baseline and after six months of treatment with pirfenidone.

## 2. Materials and Methods

### 2.1. Study Design, Population and Definitions

This prospective, observational study was a part of a larger project and included consecutive subjects with IPF who were qualified for the antifibrotic treatment. The study participants were recruited from the outpatient clinic of the Department of Internal Medicine, Pulmonary Diseases and Allergy, the Medical University of Warsaw between October 2017 and February 2019. The inclusion criteria for subjects with IPF were as follows: definite IPF diagnosis according to the 2011 ATS/ERS statement [21], forced vital capacity (FVC) > 50% of predicted, lung transfer factor for carbon monoxide (TLCO) > 30% of predicted and written informed consent to participate in the study. The exclusion criteria were: secondary cause of lung fibrosis, lung disease other than lung fibrosis, contraindications for antifibrotic therapy, prior treatment with either pirfenidone or nintedanib, malignancy, past or current treatment with systemic steroids or any immunosuppressive agent, respiratory infection within six weeks before the study onset and no written informed consent to participate in the study. The control group comprised subjects without underlying lung disease and a normal spirometry, with no history of malignancy, autoimmune diseases, renal or hepatic impairment and not using systemic steroids or any immunosuppressive agents. The major exclusion criterion for this group was respiratory infection within six weeks before the study. The study protocol was approved by the Institutional Review Board (KB/215/2017).

All IPF subjects received antifibrotic therapy as prescribed by the attending physician. Demographic data, information on smoking history, symptoms and comorbidities were collected. The GAP index and staging system were used to estimate the mortality risk [22]. Lung function tests, a six-minute walking test (6 MWT) and EBC collection were performed at baseline and after six months of treatment with pirfenidone. In the control group, the assessment was conducted only at baseline and comprised the following data: demographic data, smoking history, comorbidities and the results of lung function testing. All control subjects underwent EBC collection at baseline.

### 2.2. Exhaled Breath Condensate Collection

EBC samples were collected using the TURBO-DECCS 09 system (Medivac, Parma, Italy) according to the manufacturer’s instructions and the ERS recommendations [23]. The samples were obtained during tidal breathing (20 min) through a mouthpiece with a saliva trap and at a −5 °C condensing temperature. The respiratory samples were immediately stored at −70 °C and kept frozen until analysis.

### 2.3. Cytokine Measurements in EBC

The selection of the investigated cytokines was based on our previous pilot study performed in patients with IPF in which the expression of the following cytokines was assessed on the multiplex bead-based cytokine assay: IL-1β, -1Ra, -5, -6, -8, -10, -15, granulocyte-colony stimulating factor (G-CSF), granulocyte-macrophage colony-stimulating factor (GM-CSF), interferon-gamma (IFNγ), monocyte chemoattractant protein-1 (MCP-1), macrophage inflammatory protein 1 alpha (MIP-1α) and beta (MIP-1β), platelet-derived growth factor-beta (PDGF-β), TNFα, VEGF [24]. Of these, only five (IL-6, IL-8, IL-15, TNFα and VEGF) reached concentrations above the detection levels and were therefore selected for the current analysis. Cytokine concentrations in EBC were determined by the ELISA technique according to the manufacturer’s instructions. All measurements were performed in duplicate. We used: Human IL-6 Quantikine HS ELISA (R&D Systems, Minneapolis, MN, USA) with sensitivity 0.031 pg/mL and detection range 0.2–10 pg/mL, Human IL-8 Quantikine HS ELISA (R&D Systems, Minneapolis, MN, USA) with sensitivity 0.4 pg/mL and detection range 1–64 pg/mL, Human IL-15 Quantikine ELISA (R&D Systems, Minneapolis, MN, USA) with sensitivity 2 pg/mL and detection range 3.9–250 pg/mL, Human VEGF Quantikine ELISA (R&D Systems, Minneapolis, MN, USA) with sensitivity 9 pg/mL and detection range 15.6–1000 pg/mL, Human TNFα Quantikine ELISA (R&D Systems, Minneapolis, MN, USA) with sensitivity 0.049 pg/mL and detection range 0.2–10 pg/mL. 

### 2.4. Statistical Analysis

Continuous variables were described by median and interquartile range (IQR). Categorical variables were presented by number and percentage values. Statistical analysis was conducted using Statistica 13.3 (StatSoft Inc., Tulsa, OK, USA) software package. In the first step, basic descriptive statistics were calculated, and groups were compared with Fisher’s exact test. The differences between continuous variables were tested using the Mann–Whitney U-test for independent groups and the Wilcoxon signed-rank test for dependent groups. The strength and direction of the relationship between two variables were measured with Spearman‘s rank correlation coefficient. The level of significance in this study was assumed to be *p* < 0.05.

## 3. Results

### 3.1. Clinical Characteristics of the Study Participants and EBC Cytokine Concentrations at Baseline

Twenty-nine patients with IPF (median age 73 (68–78) years, 83% male) and 13 control subjects (median age 62 (61–76) years, 69% male) were initially enrolled. There were no differences between the groups regarding baseline demographic characteristics. The proportion of current smokers was higher in controls, but the overall burden of smoking was similar in both groups. IPF patients had a significantly lower FVC than control subjects (Table 1).

**Figure 1:**
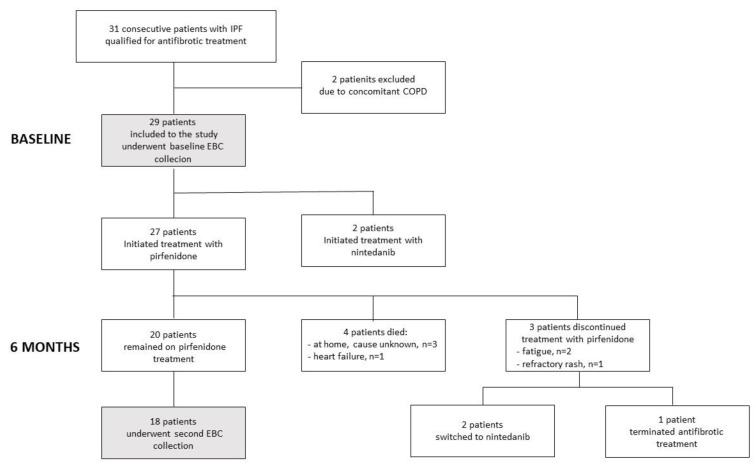
Flow chart showing enrolment and completion of the study for patients with idiopathic pulmonary fibrosis.

Twenty-seven (93.1%) patients qualified for treatment with pirfenidone and two (6.9%) for nintedanib. This disparity resulted from the fact that, in Poland, pirfenidone has been reimbursed by the National Health Fund since January 2017, while nintedanib was approved for reimbursement approximately one year later, in March 2018, and has been available in our department since July 2018. Among the 27 patients on pirfenidone, 20 continued the treatment until the follow-up after six months, four died, and three ceased pirfenidone treatment because of adverse effects (Figure 1). The two patients receiving nintedanib continued treatment throughout the investigation period with an uneventful course.

Detectable levels of IL-8 were found in 24% (7/29) and 77% (10/13) samples, of IL-6 in 79% (23/29) and 62% (8/13), and of VEGF-A in 76% (22/29) and 77% (10/13) of the samples from patients with IPF and controls respectively. A lower proportion of detectable values was found for TNF α: 10% (3/29) and 0% (0/13) for patients with IPF and controls, respectively, while the levels of IL-15 were under the limit of detection in all the analyzed EBC samples in both groups.

**Figure 2:**
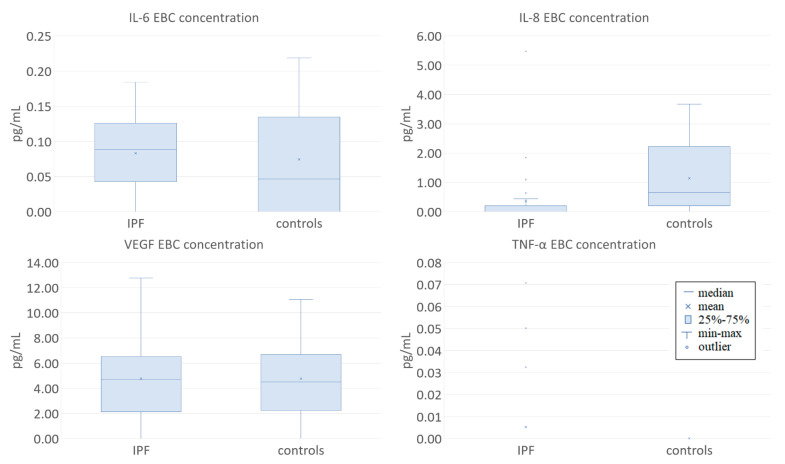
Comparison of the baseline concentrations of selected cytokines in exhaled breath condensate (EBC) from patients with IPF and controls.

The EBC concentration of IL-8 was significantly lower in patients with IPF compared to controls (0.0 (0.0–0.0) and 0.65 (0.4–1.79) pg/mL respectively, *p* = 0.005). EBC IL-6 (0.09 (0.05–0.12) and 0.05 (0.0–0.13) pg/mL respectively, *p* = 0.586) and VEGF-A (4.69 (4.27–5.96) and 4.5 (4.46–5.62) pg/mL respectively, *p* = 0.837) did not differ between these groups; the levels of TNF-α and IL-15 were not compared due to the small number of samples with detectable concentrations of these cytokines (Figure 2).

We found a negative correlation between EBC VEGF-A levels and FVC % of predicted value (r = −0.43; *p* = 0.020 and TLC % of predicted value (r = −0.54; *p* = 0.008), as well as positive correlation between EBC VEGF-A levels and the change of distance in 6MWD (m) (r = 0.71; *p* = 0.014) in patients with IPF. A positive correlation between IL-8 levels and BMI was found in both groups (r = 0.41; *p* = 0.028 for the IPF group and r = 0.62; *p* = 0.023 for the control group). We did not find any correlations between IL-6, IL-8 or VEGF-A levels and age, smoking exposure, time since smoking cessation, TLCO % of predicted value, delta SpO2 in 6MWT.

There was a negative correlation between EBC IL-6 and TNF-α, as well as a positive correlation between EBC IL-6 and VEGF-A (r = −0.36, *p* = 0.02 and r = 0.35, *p* < 0.001, respectively).

### 3.2. Clinical Characteristics of IPF Patients and EBC Cytokine Concentrations after Six Months of Treatment with Pirfenidone

Twenty patients remained on pirfenidone treatment for six months; of those, 18 (90%) had EBC collection at baseline and after six months of treatment. Except for a significantly lower BMI after six months of treatment, the basic characteristics of the patients and their lung function did not change significantly in the follow-up period (Table 2).

**Figure 3:**
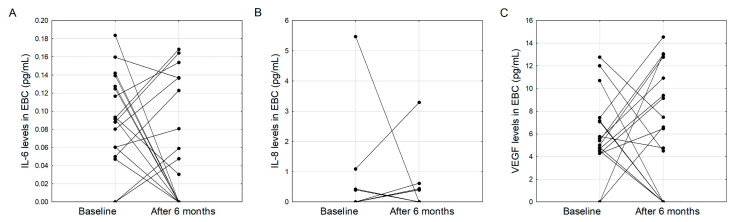
Cytokine concentrations in EBC during the six months of assessment: IL-6 (**A**), IL-8 (**B**), VEGF-A (**C**).

There were no differences between cytokine concentrations at baseline and after six months of pirfenidone treatment in patients with IPF: 0.09 (0.06–0.13) vs 0.04 (0.0–0.14) pg/mL for IL-6 (*p* = 0.184); 0.0 (0.0–0.0) vs 0.0 (0.0–0.04) pg/mL for IL-8 (*p* = 0.441), and 4.9 (4.27–7.12) vs 6.54 (0.0–10.92) pg/mL for VEGF-A (*p* = 0.653) at baseline and after six months of treatment (Figure 3).

The change in EBC VEGF-A levels correlated with the change in 6MWD (m) (r = 0.54, *p* = 0.045) and with the change of IL-6 level (r = 0.7, *p* = 0.001), but not with the change in GAP score, BMI or pulmonary lung function. We found no significant correlations between changes in EBC IL-6 and IL-8 levels and changes in GAP score, BMI, FVC, TLC, TLCO and 6MWD. 

Additional analyses were made for patients in whom EBC IL-6 and VEGF levels decreased, and those in whom an increase in these cytokines was observed after six months of treatment with pirfenidone (Figure 3A and Figure 3C respectively). Except for a greater TLC increase in those who had a higher EBC IL-6 level following treatment (4.7 (−0.1–8.98) vs. −6.5 (−13.4–−3.0) % of predicted value, *p* = 0.005) and a higher BMI after six months in those who had an increase in VEGF level (29.0 (26.1–30.3) vs. 26.0 (22.2–27.3) kg/m^2^, *p* = 0.048), no significant differences were found between the groups (Tables S1 and S2 in the Supplementary Material).

A simultaneous increase of EBC IL-6 and VEGF was found in 9/20 (45%) patients, while in seven out of 20 (35%) patients, both markers decreased after treatment. These two groups did not differ in terms of clinical and pulmonary function data (Table S3 in the Supplementary Material).

## 4. Discussion

Our study aimed to assess the changes in the EBC cytokine profile following a six-month treatment with pirfenidone in patients with IPF. We found a limited utility of ELISA measurements for IL-15 and TNFα in EBC from these patients. We showed that EBC concentrations of IL-8 are significantly lower in nontreated patients with IPF when compared to controls. Finally, we did not demonstrate significant changes in the EBC concentration of IL-6, IL-8 and VEGF-A during a six-month treatment with pirfenidone in these patients. This is one of the few studies analyzing the usefulness of EBC in monitoring treatment response to antifibrotic agents in patients with IPF and, to our knowledge, the first study to assess IL-6, IL-8, IL-15, TNF-α and VEGF-A in EBC from patients with IPF.

Although a previous report showed that EBC analysis might modestly contribute to the diagnosis and monitoring of IPF [25], some authors demonstrated that EBC composition might discriminate between IPF patients and control subjects [20,26]. It seems that these equivocal findings may result from the complex cytokine interplay in the pathogenesis of IPF and a (still) insufficient knowledge of the specific cytokines involved. Another contributing factor may be the character of EBC as a respiratory sample—although easily and noninvasively obtained, its major flaw is the high dilution of its nonvolatile components due to the high content of vapor. Therefore, EBC biomarker concentrations generally tend to be very low and may not be detectable with available testing methods [23,27]. In our study, the selection of the cytokines (IL-6, IL-8, IL-15, TNF-α and VEGF-A) was based on our previous pilot study involving patients with IPF in which the EBC cytokine profile was assessed on the Luminex platform. Not only did we show that detectable concentrations of these five cytokines were present in EBC, but we also found some significant correlations between cytokine levels and IPF clinical indices in that study [24].

In the present study, we assessed levels of these cytokines by ELISA technique. In a certain proportion of samples, they were below the assay detection limit. This particularly referred to IL-15, and TNFα as high sensitivity assays for these cytokines were unavailable, which precluded their further analysis. The comparison of the EBC cytokine concentrations between nontreated patients with IPF and controls showed a significantly higher EBC concentration of IL-8 in the latter. This is a rather unexpected finding, as it is not in line with earlier observations that almost uniformly support the involvement of IL-8 as a promoter of fibrogenesis [28,29,30]. Elevated serum concentrations of IL-8 were found in patients with IPF compared to controls by Ziora et al. [4]. Willems et al. reported higher IL-8 levels in bronchoalveolar lavage fluid (BALF) [31], and Guiot et al. reported higher sputum IL-8 in patients with IPF when compared to controls [6].

To our knowledge, there have been no reports on IL-8 levels in EBC from patients with IPF to date. Not only did we find lower EBC IL-8 in patients with IPF when compared to controls, but also IL-8 reached detectable concentrations in 24% of these patients (vs. 77% controls) despite the use of a high sensitivity assay. This indicates that our finding may not be incidental and needs confirmation in larger studies. With the missing data on IL-8 in EBC, we currently find it difficult to explain. One of the potential reasons for the discrepancy between our results and the findings of other authors may be associated with the varying biomarker concentrations in different body compartments. This has been well documented in samples from patients with chronic obstructive pulmonary disease or asthma [32,33]. Reports on mutual correlations of biomarker levels in different biological samples from patients with IPF are very scarce. In their analysis of alarmins in serum and EBC, Majewski et al. reported a weak but negative correlation between serum and EBC IL-33. Despite higher serum and EBC TSLP levels in IPF patients when compared to controls in that study, the concentrations of TSLP in these two materials did not correlate [20]. Krauss et al. also did not find relationships between EBC and BALF concentrations of PGE2 and 8-isoprostane in patients with different interstitial lung diseases, including IPF [25]. Therefore, we believe that cytokine level measurements in serum, BALF and sputum should not be extrapolated to EBC in patients with IPF. Although IL-6, -8, -15, VEGF and TNF-α were shown to contribute to the pathogenesis of IPF [7,31,34,35,36] their EBC levels in our study were either below the detection limit (IL-15 and TNFα) or did not differ significantly between patients with IPF and controls (IL-6, VEGF-A).

A six-month treatment with pirfenidone did not significantly change the EBC concentrations of IL-6, IL-8 and VEGF. This is consistent with earlier findings on other EBC cytokines [20]. There are, however, reports on antifibrotic treatment-induced changes in the cytokine profile in serum [37,38], but these did not include the cytokines investigated in the present study. Ronan et al. showed an increase in the level of IL-10 and IL-4 as well as VEGF-A but not IL-6, IL-8, IL-15 and TNF-α over a six-month treatment period with pirfenidone [18]. Despite the lack of a significant difference in the cytokine concentrations at baseline and at six months, we observed a somewhat bimodal response pattern, with some patients demonstrating a higher, and some showing lower post-treatment IL-6 and VEGF-A concentrations when compared to baseline values (Figure 3A,C). The clinical significance of this finding needs to be further investigated; nevertheless, this may indicate that response patterns to pirfenidone may not be uniform, and the concept of personalized treatment may also apply to antifibrotic therapy in patients with IPF.

We did not observe any significant changes in lung function over the treatment period what may be interpreted as a sign of disease stability. In the study by Yoshikawa et al., an FVC decline of ≥10% and/or a TLCO decline of ≥15% of baseline was observed in 34.7% of the investigated group after six to nine months of observation [38]. Majewski et al. reported a more favorable treatment response, with FVC and TLCO stability in 60.8% and 87.1% of the patients over six months of treatment with pirfenidone, respectively [39]. In our series, the median increase in FVC was 2.7% of predicted, and a decrease in TLCO was 0.8% of predicted (data not shown) and none of the study participants were excluded from further analysis because of a lung function decline surpassing the threshold of 10% of baseline FVC. Of note, we found a significant decrease in BMI after six months of treatment with pirfenidone, which is consistent with earlier findings on the adverse effects of this drug [39,40]. Despite the lack of differences in EBC VEGF-A between IPF patients and controls and between IPF patients at baseline and after six months of treatment with pirfenidone, our findings provide some new data supporting the usefulness of VEGF-A as a marker in IPF. VEGF is a potent angiogenic factor that is involved in the maintenance of the normal lung tissue and repair, with VEGF-A being most abundant in the lungs. The exact role of this glycoprotein in IPF is still obscure and may not be associated with its angiogenic properties only [36]. Nintedanib, which, among others, exhibits anti-VEGF activity, is currently one of the two compounds approved for IPF treatment with an explicitly documented antifibrotic effect [41]. However, studies on VEGF in IPF produced equivocal results. Some studies suggest the protective role of VEGF in lung fibrosis. Murray et al. showed that lung tissue and BALF VEGF was lower in patients with IPF when compared to controls, lower plasma VEGF was associated with a more progressive IPF pattern and provided evidence on the protective role of VEGF in bleomycin-induced fibrosis model [42]. Barratt et al. postulated that conflicting data on both profibrotic and protective VEGF properties might result from the different roles of VEGF-A isoforms, indicating that VEGF-A165a may enhance, whereas VEGF-A165b may inhibit fibrinogenesis [43]. In our study, EBC VEGF-A inversely correlated with FVC and TLC in nontreated patients with IPF what could indirectly support the hypothesis on its profibrotic activity. Although there was a positive correlation between the change in EBC VEGF-A levels and the change in 6MWD after six months of treatment with pirfenidone, this was not accompanied by an improvement in the absolute 6MWD and associated with a greater degree desaturation at peak exercise; therefore it seems that this finding is negligible. It seems that the analysis of the EBC cytokine profile in response to treatment with nintedanib will provide additional insight into the role of VEGF in IPF, and we will soon be able to present such preliminary data.

There are several limitations of the study that need to be acknowledged. First, the diagnostic performance of cytokine measurements in EBC was relatively low and thus precluded all the planned analyses. However, our study was also exploratory, and one of our aims was to assess the feasibility of the selected cytokine measurements in EBC. Our findings confirm that conducting research with the use of EBC is challenging and highlight the need for high sensitivity measurement methods for EBC analysis. This may be the plausible explanation for the low diagnostic performance of the applied ELISA kits for IL-15 and TNF-α in our cohort as we did not use high sensitivity assays for these cytokines—to our knowledge such assays were not commercially available at the time of the study. Due to the particularly small sample volumes (as with EBC) no measurements were performed to normalize concentrations for dilution between subjects.

On the other hand, this was also the case with VEGF-A and not only detectable concentrations of this marker were found in a considerable proportion of the investigated subjects but also some significant correlations between EBC VEGF-A and IPF clinical data were demonstrated. The second important limitation of this study is the disproportion between the number of patients treated with pirfenidone and those treated with nintedanib. This, however, was a result of the initial inaccessibility to treatment with nintedanib during the investigation period caused by the internal regulations of the Polish National Health Fund and the time interval between approval for the reimbursement of pirfenidone and nintedanib for patients with IPF. Therefore, to assure the maximal homogeneity of the investigated group and the credibility of our results, the analysis of post-treatment data was limited to patients treated with pirfenidone.

Some differences in the characteristics of the investigated groups may also be regarded a drawback. Subjects from the control group tended to be younger; this was a result of significant difficulties in the recruitment of elderly subjects without lung disease who would voluntarily undergo respiratory investigations for research purposes. The difference in smoking status was negligible as the number of packyears did not differ between the groups. The differences in the incidence of comorbidities may result from the higher morbidity in IPF patients as compared to the general population [44,45], therefore our results reflect real-life conditions.

To conclude, our findings provided some new data supporting the potential usefulness of VEGF-A as a marker in IPF. A six-month treatment with pirfenidone was not associated with a significant change in the tested cytokine profile of EBC. The low EBC IL-8 level in patients with IPF is a novel finding which needs confirmation in larger studies. 

## Figures and Tables

**Table 1:** Baseline characteristics of the study participants.

	IPF *n* = 29	Controls *n* = 13	*p*
Male gender	24 (83%)	9 (69%)	0.323
Age (years)	73 (68–78)	62 (61–76)	0.058
BMI (kg/m2)	28.2 (25.8–29.7)	27.9 (25.2–30.1)	0.747
Months since IPF diagnosis	24 (13–37)	NA	-
Months since first symptoms	23 (10–40)	NA	-
GAP score (points)	4 (2–4)	NA	-
GAP stage I/II/III (number)	12/17/0		-
Lung function			
FVC (% of predicted)	81.6 (70.7–101.2)	119.0 (97.0–123.0)	<0.001
TLC (% of predicted)	79.3 (71.0–94.5)	NA	-
TLCO (% of predicted)	57.7 (40.0–70.5)	NA	-
6 MWD (meters)	482.5 (420–520)	NA	-
Δ SpO_2_ in 6 MWT (%)	9.5 (5–12.5)	NA	-
Smoking status			
Current/ex/never smoker (number)	0/25/4	2/7/4	0.029
Time since smoking cessation (years)	23 (15–31)	28 (20–45)	0.296
Overall smoking exposure (pack-years)	20 (12–47)	10 (5–20)	0.086
Comorbidities *n* (%)			
Diabetes mellitus	12 (41%)	1 (8%)	0.024
Arterial hypertension	14 (48%)	6 (46%)	0.899
Ischemic heart disease	12 (41%)	2 (15%)	0.323
Cardiac arrhythmias	1 (3%)	3 (23%)	0.045
GERD	16 (55%)	0	<0.001

Data are presented as median and interquartile range or number and percentage. Abbreviations: IPF—idiopathic pulmonary fibrosis, BMI—body mass index, GERD—gastroesophageal reflux disease, GAP—gender, age, physiology prognostic index for IPF [22], FVC—forced vital capacity, TLC—total lung capacity, TLCO—lung transfer factor for carbon monoxide, 6 MWD—six-minute walk distance, ΔSpO_2_ in 6 MWT—the difference between blood oxygenation at baseline and at peak exercise in the six-minute walk test, NA—not applicable.

**Table 2:** Comparison of the basic characteristics of the investigated patients at baseline and after six months of treatment with pirfenidone (*n* = 20).

	At Baseline	After Six Months of Pirfenidone Treatment	*p*
BMI (kg/m^2^)	28.2 (26.0–29.6)	26.5 (22.9–29.0)	0.024
FVC (% of predicted)	81.4 (66.4–101.9)	82.5 (67.9–103.7)	0.393
TLC (% of predicted)	79.3 (71.4–90.3)	71.1 (67.9–81.5)	0.277
TLCO (% of predicted)	57.7 (40.0–70.5)	52.2 (39.2–70.1)	0.974
6 MWD (meters)	485.0 (420.0–530.0)	482.5 (430.0–540.0)	0.958
ΔSpO_2_ in 6 MWT	8.5 (5.5–11.5)	9.0 (7.0–13.0)	0.793

Data are presented as median (interquartile range). Abbreviations: BMI—body mass index, FVC—forced vital capacity, TLC—total lung capacity, TLCO—transfer factor of the lung for carbon monoxide, 6 MWD—six-minute walk distance, 6 MWT—six-minute walk test, ΔSpO_2_—change in oxygen saturation.

## Supplementary Materials

The following are available online at https://www.mdpi.com/2077-0383/9/8/2523/s1, Table S1: Clinical data of patients with IPF with exhaled breath condensate IL-6 concentration decrease and increase following six months of treatment with pirfenidone, Table S2: Clinical data of patients with IPF with exhaled breath condensate VEGF-A concentration decrease and increase following six months of treatment with pirfenidone, Table S3: Comparison of clinical data of patients with IPF who demonstrated simultaneous IL-6 and VEGF-A increase and those who showed simultaneous IL-6 and VEGF-A decrease in EBC following six months of treatment with pirfenidone.
