# Supplementary material for: Exhaled Biomarkers in Idiopathic Pulmonary Fibrosis—A Six-Month Follow-up Study in Patients Treated with Pirfenidone
Source: J Clin Med. 2020 Aug 5;9(8):2523. doi: 10.3390/jcm9082523 (PMC7465603; doi:10.3390/jcm9082523)
Supplement: Supplementary file 1 [file jcm-09-02523-s001.pdf]

Table S1. Clinical data of patients with IPF with exhaled breath condensate IL–6 concentration decrease and increase following six months of treatment with pirfenidone.

		**Lower EBC IL–6 at 6 Months*** ***N*** **= 10**	**Higher EBC IL–6 at 6 Months*** ***N*** **= 8**	***p***
Male gender		10 (100%)	6 (75%)	0.094
Age (years)		71.5 (69–79)	76.5 (69.5–77.5)	0.562
Months since IPF diagnosis		17 (13–55)	33.5 (15.5–51.5)	0.756
Months since first symptoms		27 (12–60)	16 (6–59.5)	0.657
GAP score (points)		3.5 (2–5)	4 (3–4)	0.722
GAP stage I/II/III (number)		4/6/0	3/5/0	0.914
BMI (kg/m^2)	baseline	27.65 (26.2–29.5)	28.88 (28.15–29.85)	0.307
	at 6 months	26.8 (23.4–28.7)	26.7 (22.9–29.9)	0.986
**Lung function**				
FVC (% of predicted)	baseline	83.8 (67.2–102.5)	77.8 (63.7–96)	0.775
	at 6 months	82.5 (71.6–106.3)	80.2 (62.2–100)	0.749
Δ FVC (% of predicted)		2.85 (1.3–5)	2.15 (−1.45 – 3.45)	0.505
TLC (% of predicted)	baseline	84.85 (78.2–91.2)	70.2 (65–72.2)	0.140
	at 6 months	70.6 (67.9–81.3)	77.95 (70.3–81.6)	0.464
Δ TLC (% of predicted)		−6.5 (−13.4 – −3)	4.7 (−0.1–9)	0.005
TLCO (% of predicted)	baseline	48.3 (36.7–70.5)	57.8 (40–76.2)	0.599
	at 6 months	48.7 (35.6–63.6)	50.8 (39.2–73.2)	0.815
Δ TLCO (% of predicted)		−1.1 (−5.3–3.15)	−0.9 (−3–3.5)	0.569
6 MWD (meters)	baseline	520 (485–545)	435 (420–505)	0.219
	at 6 months	482.5 (437.5–530)	480 (430–540)	0.837
Δ MWD (meters)		−7.5 (−60–5)	12.5 (−15–45)	0.267
Δ SpO2 in 6 MWT (%)	baseline	8.5 (6.5–10.5)	8 (5–16)	0.443
	at 6 months	9 (6.5–11.5)	8.5 (7–14)	0.535

*Compared to EBC concentration at baseline. Data are presented as median and interquartile range or number and percentage. Abbreviations: IPF – idiopathic pulmonary fibrosis, GAP – gender, age, physiology prognostic index for IPF, BMI – body mass index, , FVC – forced vital capacity, TLC – total lung capacity, TLCO – lung transfer factor for carbon monoxide, 6 MWD – 6–minute walk distance, ΔSpO2 in 6 MWT – the difference between blood oxygenation at baseline and at peak exercise in the 6–minute walk test.

Table S2. Clinical data of patients with IPF with exhaled breath condensate VEGF–A concentration decrease and increase following six months of treatment with pirfenidone.

		Lower EBC VEGF–A at 6 Months* $N = 9$	Higher EBC VEGF–A at 6 Months* $N = 8$	p
Male gender		8 (89%)	8 (100%)	1.000
Age (years)		71 (69–79)	76 (70–77)	0.555
Months since IPF diagnosis		16 (13–55)	30 (17–39)	0.596
Months since first symptoms		24 (12–36)	23 (12–66)	0.825
GAP score (points)		4 (2–5)	4 (3–4)	0.964
GAP stage I/II/III (number)		3/6/0	4/5/0	0.629
BMI (kg/m^2)	baseline	27.3 (26.2–28.2)	29 (28.4–30)	0.077
	at 6 months	26 (22.2–27.3)	29.01 (26.11–30.25)	0.048
Lung function				
FVC (% of predicted)	baseline	77.5 (67.2–87.7)	83.8 (74.4–108.2)	0.529
	at 6 months	82.5 (68.5–84.4)	85.4 (75–113.5)	0.565
Δ FVC (% of predicted)		2.85 (1.3–5)	2.8 (1.3–3.8)	0.789
TLC (% of predicted)	baseline	84.5 (73.6–87.8)	72.2 (65–94.5)	0.525
	at 6 months	70.85 (66.7–79)	79.2 (70.3–92.1)	0.257
Δ TLC (% of predicted)		−6.15 (–3.4 – –3)	−1.9 (–2.4 – –1.7)	0.206
TLCO (% of predicted)	baseline	43.3 (36.35–65.85)	61.65 (50.05–73.35)	0.217
	at 6 months	44.45 (35.45–68.4)	52.2 (43.95–67.95)	0.827
Δ TLCO (% of predicted)		–0.8 (–2.8–5.9)	–1 (–4.2–0.4)	0.779
6 MWD (meters)	baseline	485 (420–550)	480 (427.5–530)	0.837
	at 6 months	505 (390–555)	460 (450–540)	0.702
Δ MWD (meters)		0 (–20–5)	10 (–60–35)	0.724
Δ SpO2 in 6 MWT (%)	baseline	9 (5–13)	8 (6–11)	0.742
	at 6 months	10 (7–13)	7 (3–10)	0.188

*Compared to EBC concentration at baseline. Data are presented as median and interquartile range or number and percentage. Abbreviations: IPF – idiopathic pulmonary fibrosis, GAP – gender, age, physiology prognostic index for IPF, BMI – body mass index, , FVC – forced vital capacity, TLC – total lung capacity, TLCO – lung transfer factor for carbon monoxide, 6 MWD – 6–minute walk distance, Δ SpO2 in 6 MWT – the difference between blood oxygenation at baseline and at peak exercise in the 6–minute walk test.

Table S3. Comparison of clinical data of patients with IPF who demonstrated simultaneous IL–6 and VEGF–A increase and those who showed simultaneous IL–6 and VEGF–A decrease in EBC following six months of treatment with pirfenidone.

		**Increase of Both EBC IL–6 and VEGF After 6 Months*** ***N* = 7**	**Decrease of Both EBC IL–6 and VEGF After 6 Months*** ***N* = 9**	***p***
Male gender		6 (86%)	8 (89%)	0.987
Age (years)		77 (68–78)	71 (69–79)	0.791
Months since IPF diagnosis		30 (10–39)	16 (13–55)	0.916
Months since first symptoms		14 (0–66)	24 (12–36)	0.657
GAP score (points)		4 (3–4)	4 (2–5)	1.0
GAP stage I/II/III (number)		3/4/0	3/6/0	0.966
BMI (kg/m^2)	baseline	29 (28.1–30)	27.3 (26.2–28.2)	0.129
	at 6 months	28 (25.5–29.9)	26 (22.2–27.3)	0.154
Lung function				
FVC (% of predicted)	baseline	81.2 (56.6–108.2)	77.5 (67.2–87.7)	0.773
	at 6 months	85.4 (58.2.6–113.5)	82.5 (68.5–84.4)	0.745
Δ FVC (% of predicted)		2.7 (0.6–4.2)	2.8 (1.3–3.8)	0.918
TLC (% of predicted)	baseline	71.35 (65–72.2)	84.5 (73.6–87.7)	0.367
	at 6 months	79.2 (70.3–81.6)	70.85 (66.7–79)	0.334
Δ TLC (% of predicted)		4.7 (−1.9–10.25)	−6.15 (−13.4–3)	0.130
TLCO (% of predicted)	baseline	61.65 (51.8–76.2)	43.3 (36.35–65.85)	0.250
	at 6 months	52.2 (39.2–73.2)	44.45 (35.45–68.4)	0.926
Δ TLCO (% of predicted)		−1 (−3– −0.8)	−0.8 (−2.8–5.9)	0.808
6 MWD (meters)	baseline	445 (420–505)	485 (420–550)	0.584
	at 6 months	510 (450–540)	505 (390–555)	0.560
Δ MWD (meters)		15 (0–55)	0 (−20–5)	0.204
Δ SpO_2 in 6 MWT (%)	baseline	7.5 (5–11)	9 (5–13)	0.712
	at 6 months	7 (7–10)	10 (7–13)	0.313

*Compared to EBC concentration at baseline. Data are presented as median and interquartile range or number and percentage. Abbreviations: IPF – idiopathic pulmonary fibrosis, GAP – gender, age, physiology prognostic index for IPF, BMI – body mass index, , FVC – forced vital capacity, TLC – total lung capacity, TLCO – lung transfer factor for carbon monoxide, 6 MWD – 6–minute walk distance, ΔSpO_2 in 6 MWT – the difference between blood oxygenation at baseline and at peak exercise in the 6–minute walk test.
